# A Comparison of Midwife-Led and Medical-Led Models of Care and Their Relationship to Adverse Fetal and Neonatal Outcomes: A Retrospective Cohort Study in New Zealand

**DOI:** 10.1371/journal.pmed.1002134

**Published:** 2016-09-27

**Authors:** Ellie Wernham, Jason Gurney, James Stanley, Lis Ellison-Loschmann, Diana Sarfati

**Affiliations:** 1 Department of Public Health, University of Otago, Wellington, New Zealand; 2 Centre for Public Health Research, Massey University, Wellington, New Zealand; University of Manchester, UNITED KINGDOM

## Abstract

**Background:**

Internationally, a typical model of maternity care is a medically led system with varying levels of midwifery input. New Zealand has a midwife-led model of care, and there are movements in other countries to adopt such a system. There is a paucity of systemic evaluation that formally investigates safety-related outcomes in relationship to midwife-led care within an entire maternity service. The main objective of this study was to compare major adverse perinatal outcomes between midwife-led and medical-led maternity care in New Zealand.

**Methods and Findings:**

This was a population-based retrospective cohort study. Participants were mother/baby pairs for all 244,047 singleton, term deliveries occurring between 1 January 2008 and 31 December 2012 in New Zealand in which no major fetal, neonatal, chromosomal or metabolic abnormality was identified and the mother was first registered with a midwife, obstetrician, or general practitioner as lead maternity carer. Main outcome measures were low Apgar score at five min, intrauterine hypoxia, birth-related asphyxia, neonatal encephalopathy, small for gestational age (as a negative control), and mortality outcomes (perinatal related mortality, stillbirth, and neonatal mortality). Logistic regression models were fitted, with crude and adjusted odds ratios (ORs) generated for each outcome for midwife-led versus medical-led care (based on lead maternity carer at first registration) with 95% confidence intervals. Fully adjusted models included age, ethnicity, deprivation, trimester of registration, parity, smoking, body mass index (BMI), and pre-existing diabetes and/or hypertension in the model. Of the 244,047 pregnancies included in the study, 223,385 (91.5%) were first registered with a midwife lead maternity carer, and 20,662 (8.5%) with a medical lead maternity carer. Adjusted ORs showed that medical-led births were associated with lower odds of an Apgar score of less than seven at 5 min (OR 0.52; 95% confidence interval 0.43–0.64), intrauterine hypoxia (OR 0.79; 0.62–1.02), birth-related asphyxia (OR 0.45; 0.32–0.62), and neonatal encephalopathy (OR 0.61; 0.38–0.97). No association was found between lead carer at first registration and being small for gestational age (SGA), which was included as a negative control (OR 1.00; 0.95–1.05). It was not possible to definitively determine whether one model of care was associated with fewer infant deaths, with ORs for the medical-led model compared with the midwife-led model being 0.80 (0.54–1.19) for perinatal related mortality, 0.86 (0.55–1.34) for stillbirth, and 0.62 (0.25–1.53) for neonatal mortality. Major limitations were related to the use of routine data in which some variables lacked detail; for example, we were unable to differentiate the midwife-led group into those who had received medical input during pregnancy and those who had not.

**Conclusions:**

There is an unexplained excess of adverse events in midwife-led deliveries in New Zealand where midwives practice autonomously. The findings are of concern and demonstrate a need for further research that specifically investigates the reasons for the apparent excess of adverse outcomes in mothers with midwife-led care. These findings should be interpreted in the context of New Zealand’s internationally comparable birth outcomes and in the context of research that supports the many benefits of midwife-led care, such as greater patient satisfaction and lower intervention rates.

## Abstract 2

In a retrospective study of routinely collected medical data, Ellie Wernham and colleagues compare adverse fetal and neonatal outcomes associated with midwife-led and medical-led models of care.

## Author Summary

**Why Was This Study Done?:**

- New Zealand adopted an autonomous midwife-led model of maternity care in 1990. There has never been a detailed review examining what effect, if any, midwife-led care has on adverse outcomes for unborn and newly born infants in the New Zealand setting.
- A review of the safety of New Zealand’s midwife-led maternity system is of relevance to other countries that are considering adopting this model of care.

**What Did the Researchers Do and Find?:**

- We examined data on all full-term births in which no serious abnormalities were detected in the baby that occurred in New Zealand over a 5-y period (total sample size 244,047).
- We compared rates of adverse outcomes for unborn and newly born infants among women who were in midwife-led versus medical-led care at first registration during antenatal care.
- Overall rates of adverse outcomes in the New Zealand setting were low and comparable to international rates.
- We found that, among mothers with medical-led care compared with midwife-led care, there were lower odds of some adverse outcomes for infants. These included oxygen deprivation during the delivery (birth-related asphyxia) (55% lower odds), neonatal encephalopathy—a condition that can result in brain injury (39% lower odds), and low Apgar score, which is a measure of infant well-being immediately postdelivery, with a low score being indicative of an unwell baby (48% lower odds).

**What Do These Findings Mean?:**

- Despite New Zealand having overall internationally comparable maternity outcomes, the findings of this study suggest that avoidable adverse outcomes may still be occurring.
- Further research that examines the potential reasons for an apparent excess in adverse outcomes in midwife-led care is required.

## Introduction

The organization of maternity systems varies internationally. Most systems involve a level of collaboration between medical and midwifery practitioners. Many countries have medical-led maternity care systems in which midwives provide a significant amount of care but are not autonomous in their practice [1]. However, in two countries, the Netherlands and New Zealand, midwife-led continuity of care is the typical model and has been defined as one in which “the midwife is the lead professional in the planning, organization and delivery of care given to a woman from initial booking to the postnatal period” [2]. This definition is consistent with how midwife-led care operates in New Zealand. There are movements in other countries to adopt such a system [3–5].

New Zealand’s maternity system experienced major reform in 1990 when legislation resulted in substantial changes to the way maternity care was funded, allowing for complete midwifery autonomy and removal of the requirement for a nursing background for midwives (i.e., direct-entry education) [6]. As a result, currently four out of five mothers have a midwife as their lead maternity carer [7]. For these women, a doctor is only generally involved if the mother has or develops obstetric risk factors. In New Zealand, each care provider type is paid (by the New Zealand government) equally based on the services provided; only medical-led carers are able to charge fees on top of the government subsidy. Thus, midwifery care is provided at no cost to the patient. Obstetric-led care is substantially less frequent than midwife-led care (5.5% versus 81.1% in 2012) [7]. Obstetricians tend to have two distinct subgroups of patients: those identified as high risk, for whom obstetric care is provided at no cost (by hospital-employed practitioners), and those who choose private fee-for-service obstetric care, which while subsidized, still comes at a substantial cost to the patient. General practitioner (GP) obstetricians, who prior to 1990 were the most common lead maternity carers, are now increasingly rare (1.1% in 2012) [7]. The lead maternity carer is responsible for overseeing and carrying out all aspects of maternity care. If the care required falls outside of the care provider’s scope of practice, it is their responsibility to refer the woman to an appropriate practitioner; however, the original care provider typically remains as the lead maternity carer in a shared care arrangement. Thus, women with a midwife as their lead maternity carer may have any level of medical input into their care, and those with an obstetrician are also likely to receive midwifery support. The changes to the maternity system in New Zealand occurred rapidly, and there has been little in the way of systematic evaluation that specifically investigates safety-related outcomes within the new system.

A 2016 Cochrane systematic review on 17,642 births across four countries reported numerous benefits associated with midwife-led models of care compared to other (standard) models of care [2]. Benefits included less fetal and neonatal loss, fewer preterm births (before 37-wk gestation), fewer interventions such as regional anesthesia and instrumental delivery, a higher chance of being attended at birth by a known midwife, and a higher chance of vaginal delivery. The Cochrane group reported no differences in rates of adverse outcomes that included neonatal convulsions, a 5 min Apgar score of less than or equal to seven, and neonatal admission to an intensive care unit [2]. However, the midwife-led interventions assessed in this review were heterogeneous, in many cases comparing highly coordinated care with routine care. Further, the midwife-led interventions frequently involved routine medical input. It was also noteworthy that in the majority of studies, the midwife-led interventions were carried out by a small number of midwives (in most cases ten or fewer). It is thus difficult to generalize the findings of this review to a population-based setting with completely autonomous midwives.

This study aimed to investigate whether the frequency of adverse perinatal outcomes differed between midwife-led and medical-led births within the New Zealand setting. The specific outcomes we investigated included mortality outcomes (perinatal related mortality, which includes stillbirth and neonatal mortality); morbidity outcomes, particularly those associated with perinatal care (Apgar score of less than seven at 5 min, intrauterine hypoxia, birth related asphyxia, and neonatal encephalopathy); and small for gestational age (SGA) as a negative control outcome.

## Methods

This was a population-based retrospective cohort study in which mother/baby pairs were identified from routinely collected maternity (live-born births) and mortality (stillborn births) data and followed up for mortality and morbidity outcomes using mortality and hospitalization data.

The study was granted ethical approval by New Zealand’s Multi-Region Ethics Committee (MEC/11/EXP/131). All data were nonidentifiable, and individual informed consent from participants was not required.

This study is reported as per Strengthening the Reporting of Observational Studies in Epidemiology (STROBE) guidelines (S1 Checklist).

### Data Sources

Maternity data are routinely collected in New Zealand from maternity claim forms submitted by lead maternity carers. Data include information on maternal characteristics (including body mass index [BMI] and smoking status at registration), obstetric characteristics (including parity, gestation at delivery, and mode of delivery), service provision (including lead maternity care type at first registration and trimester of registration), and state of the neonate at birth (including Apgar scores) [8]. These data are routinely linked with data from the National Minimum Dataset (NMDS), which records hospital discharge data. The NMDS includes demographic data and diagnostic and procedural coded data based on the International Statistical Classification of Diseases and Related Health Problems for both mother and baby [9]. These data include details of maternal conditions such as pre-existing hypertension and diabetes, fetal/neonatal abnormalities, and peri- and postnatal diagnoses including intrauterine hypoxia, birth-related asphyxia, and neonatal encephalopathy. The mortality dataset holds data on all deaths, including stillbirths, occurring in New Zealand [10].

### Study Population

Participants were included in the study if the delivery occurred within the study period of 1January 2008 to 31 December 2012, the longest time period in which all data were available. Participants met the following inclusion criteria: a singleton pregnancy, gestation equal to or greater than 37 wk, and no identification of major fetal or neonatal congenital, chromosomal, or metabolic abnormalities. These criteria were designed to exclude pregnancies with high rates of unavoidable adverse outcomes not related to care. Because our aim was to compare midwife-led and medical-led births, only women registered with either a midwife, obstetrician, or GP as their lead maternity carer were included. Those with no or an unknown lead maternity carer, a hospital as lead maternity carer (whereby the mother receives care from a team of maternity care providers working within a hospital setting), or those who registered with a lead maternity carer after the delivery were excluded.

Fetal and neonatal abnormalities were categorized based on identification of specific ICD-10-AM diagnostic codes that are included in the NMDS. The list of ICD-10-AM codes was defined by a maternal fetal medicine specialist without reference to study data (thus, exclusions were made blinded to exposure and outcomes). Codes were considered relevant if the condition increased the risk of fetal or neonatal mortality (such as anencephaly) or when the condition was considered serious in that tertiary-level neonatal admission would likely be required (such as a heart defect).

### Exposure Variables

Exposure groups were based on the participant’s lead maternity carer at first registration. Those with a midwife as their registered lead maternity carer were allocated to the midwife-led group, and those with either an obstetrician or GP as lead maternity carer were allocated to the medical-led group.

### Outcome Variables

Three types of variables were assessed: (1) mortality outcomes, (2) morbidity outcomes, and (3) SGA. These were defined as follows.

#### Mortality outcomes

Stillbirth was defined as when the infant was born deceased. Antepartum and intrapartum stillbirths could not be differentiated.

Neonatal mortality was defined as when the infant was born alive and died before the completion of the 27th day of life.

Perinatal related mortality (PRM) was defined as a composite of stillbirths and neonatal mortalities.

#### Morbidity outcomes

A low Apgar score was defined as when the Apgar score at 5 min postdelivery was recorded as lower than seven. The Apgar score is routinely recorded on the lead maternity carer (LMC) claim form. A low Apgar score at 5 min is associated with increased risk of neonatal mortality and neurological impairment [11].

Intrauterine hypoxia (ICD-10-AM codes P200, P201, and P209), birth-related asphyxia (ICD-10-AM codes P210, P211, and P219), and neonatal encephalopathy (ICD-10-AM code P9181) were identified using ICD-10-AM codes recorded in the NMDS database. These conditions are all indicative of oxygen deprivation during the antepartum period or during labor and delivery and can lead to brain injury, cerebral palsy, neurodevelopmental delay, visual or hearing impairment, and death [12]. These outcomes were assessed both individually and as a single combined outcome.

#### SGA

SGA was defined as when the birth weight was recorded as below the tenth percentile for infants born at a given gestational age (in whole weeks, as determined from our data; see S3 Table). This variable was included as a negative control outcome in order to determine whether adjustment for confounding for the main outcomes was likely to have been sufficient [13]. Being SGA is unlikely to be influenced by the model of maternity care and clearly cannot be influenced by intrapartum care. However, because the risk factors for SGA and other poor outcomes are similar, the association between model of care and SGA is likely to be affected by the same sources of confounding. If results show an association between model of care and being SGA, after adjusting for confounders, this would suggest that residual confounding has occurred.

### Additional Variables

Variables were chosen based on a priori knowledge of their plausible associations with the exposures and outcomes measured in this study. A simplified directed acyclic graph is provided (S1 Fig).

Age was calculated on the basis of age at the time of delivery. BMI was recorded on the lead maternity carer claim form, with height and weight as measured by the lead maternity carer or reported by the mother. For both these variables, there is a potential U-shaped association with adverse outcomes in which both very young or underweight and older or overweight mothers may have higher risk, so these variables were treated in two ways in multivariable models: as categorical variables (age categories <20 y, 20–35 y, and >35 y, BMI categories <19, 19–24, and ≥25) and as continuous variables. In both cases, the results of the study were almost identical. In descriptive analyses, these variables were treated as categorized variables, whilst in multivariable models, both BMI and age were treated as continuous covariates.

Deprivation was based on the mother’s place of residence using the New Zealand Deprivation Index (NZDep 2006), a small-area-based index calculated using aggregated census data based on residents’ socioeconomic characteristics (such as benefit receipt, earning under an income threshold, and access to car or phone) [14]. Scores were grouped into deciles and treated as continuous in models.

Ethnicity was self-nominated by mothers and derived from the maternity dataset. When more than one ethnic group was nominated, women were assigned according to standard prioritization procedures in the following order: Māori, Pacific, Asian (Indian), Other, and European [15]. Indian would normally be included in the Asian group, but because of their higher risk of adverse outcomes, we treated them as a separate ethnic group.

Smoking status (yes/no), parity, and trimester at registration were recorded on the LMC claim form at the time of registration.

The presence of pre-existing diabetes and/or hypertension were based on ICD-10-AM diagnostic codes recorded in the NMDS. These codes included all ICD-10-AM codes that identify the condition pre-existing to pregnancy—e.g., code “O240” for pre-existing diabetes mellitus, Type 1, in pregnancy and code “O100” for pre-existing essential hypertension complicating pregnancy, childbirth, and the puerperium.

Data on gestation at delivery and delivery type were also collected, but these were not included in models because they are considered potential mediators between the model of care and perinatal outcomes (S1 Fig).

In all cases, those with missing data (missing data were <1% for each covariate) were excluded from the multivariable analyses. This led to a complete case analysis that excluded 1.3% of pregnancies.

### Statistical Analysis

Prevalence estimates of maternal and obstetric variables for the overall cohort and for each LMC exposure group were calculated and compared. For the exposure groups, crude and age-standardized prevalence estimates were calculated with prevalence standardized to the age structure of the total study population. The Cochrane-Mantel-Haenzel statistic (*p*-value) was used to compare the distribution of variables between exposure groups, adjusted for age.

Rates of PRM and stillbirth were calculated for the total population and for each exposure group, with the total number of births in the relevant population as the denominator. For all other outcomes, pregnancies in which a stillbirth occurred were excluded from the denominator. For low Apgar score and being SGA, those with missing outcome data were excluded (both >90% complete). Logistic regression models were fitted to assess the crude associations of mother’s age (<20, 20–35, and >35 y), deprivation (deciles 1–2; 3–4; 5–6; 7–8, and 9–10), mother’s ethnicity (European, Māori, Pacific, Asian, Indian, and Other), BMI (<19;19–24; 25+), smoking status at first LMC registration (yes/no), parity (nulliparous versus other), trimester of LMC registration (trimester one versus other), and the presence of pre-existing diabetes and/or hypertension (yes/no) with each outcome. Adjusted odds ratios (ORs) were calculated for each variable by fitting further logistic models for each outcome, with each model containing all of the other confounding variables listed above. Hypothesis tests for each factor are Type III tests from the adjusted logistic regression model, testing for overall association of each given factor with the outcome (e.g., any differences in perinatal mortality outcome by age group.)

Finally, logistic regression models were fitted to calculate the odds of each outcome relative to the model of maternity care at first registration, with midwife-led care as the reference group. Crude ORs for the model of maternity care were calculated, followed by adjusted ORs in which models included age (continuous), ethnicity (European, Māori, Pacific, Asian, Indian, and other), deprivation (continuous deciles), BMI (continuous), smoking at registration (yes/no), parity (nulliparous and other), trimester of registration (trimester one and other), and pre-existing conditions (yes/no). Reported *p*-values are Type III hypothesis tests from the adjusted logistic regression models (as noted above). To account for the potential impact of clustering by region with associated potential differences in services available in different hospitals, we conducted supplementary analysis examining the above birth outcomes by model of maternity care. These models adjusted for the potential confounders as specified above and included District Health Board of residence as a random intercept term (using a generalized linear mixed model).

All data analysis was performed using SAS statistical software package version 9.3.

We were not able to further stratify the midwife-led group into those who had received medical input during pregnancy from those who had not because of data limitations. We were also not able differentiate stillbirths into antepartum and intrapartum deaths, which may have been helpful in that intrapartum deaths are more likely to be related to model of care. All analytical and other data management steps relating to inclusions and exposure categorizations were done prior to linking the data on model of care and covariates to the outcome data.

## Results

There were 286,572 singleton, term pregnancies in New Zealand in the period of the study. Of these, 3,766 were excluded because the baby was identified as having a serious congenital anomaly, and 37,691 were excluded because their LMC was not a midwife, GP, or obstetrician. Of these births without a midwife or medical LMC, 99% were explicitly noted as not having an LMC (data field entered as “No LMC,” so the mother likely had no antenatal care and presented in labor), with the remainder having a hospital or District Health Board as their registered LMC. Only 11 mothers had missing LMC data. An additional 1,068 were excluded because they registered with their LMC after delivery of the baby, in total leaving 244,047 pregnancies meeting the inclusion criteria. Of these women, 223,385 (91.5%) were first registered with a midwife as their LMC, and 20,662 (8.5%) with a medical LMC.

Table 1 shows the characteristics of mothers overall and for mothers with midwife-led and medical-led care. Compared with the medical-led group, the midwife-led group had greater proportions of younger mothers, mothers of a non-European ethnicity, and mothers from higher deprivation areas. They were also more often overweight, registered with their carer later in pregnancy, and were more often smokers. Compared with the midwife-led group, the medical-led group were more likely to be older, nulliparous, and to deliver their babies at earlier gestations. Rates of pre-existing diabetes and/or hypertension were similar between groups (0.7% for midwife-led and 0.9% for medical-led) (Table 1). Missing data were relatively rare for any given variable (<1%).

**Table 1:** Characteristics of the overall cohort and the midwife-led and medical-led groups.

Maternal Characteristic	Overall	Midwife-Led		Medical-Led		
	*n* (%)	*n* (%)	Age Standardized (%)	*n* (%)	Age Standardized (%)	*p*-Value
**Age**						<0.001
<20 y	16,380 (6.7)	15,842 (7.1)	–	538 (2.6)	–	–
20 to 35 y	187,156 (76.7)	173,623 (77.7)	–	13,533 (65.5)	–	–
>35 y	40,502 (16.6)	33,912 (15.2)	–	6,590 (31.9)	–	–
Missing data	9 (0.0)	8 (0.0)	–	1 (0.0)	–	–
**Ethnicity**						<0.001
European	132,159 (54.2)	117,547 (52.6)	53.1	14,612 (70.7)	65.7	–
Māori	60,985 (25.0)	58,902 (26.4)	25.9	2,083 (10.1)	14.8	–
Pacific	21,308 (8.7)	20,498 (9.2)	9.1	810 (3.9)	4.6	–
Asian	18,660 (7.7)	16,590 (7.4)	7.5	2,070 (10.0)	9.6	–
Indian	6,741 (2.8)	6,012 (2.7)	2.7	729 (3.5)	3.6	–
Other	4,163 (1.7)	3,806 (1.7)	1.7	357 (1.7)	1.7	–
Missing data	31 (0.0)	30 (0.0)	–	1 (0.0)	–	–
**NZ Deprivation Decile**						<0.001
1 to 2	37,191 (15.2)	31,542 (14.1)	14.4	5,649 (27.3)	22.9	–
3 to 4	41,298 (16.9)	36,809 (16.5)	16.7	4,489 (21.7)	19.4	–
5 to 6	48,348 (19.8)	43,949 (19.7)	19.8	4,399 (21.3)	21.8	–
7 to 8	56,065 (23.0)	52,250 (23.4)	23.3	3,815 (18.5)	22.4	–
9 to 10	60,638 (24.9)	58,344 (26.1)	25.9	2,294 (11.1)	13.5	–
Missing data	507 (0.2)	491 (0.2)	–	16 (0.1)	–	–
**Parity**						<0.001
Nulliparous	100,168 (41)	90,798 (40.6)	40.0	9,370 (45.3)	51.2	–
Prima or multiparous	143,858 (59)	132,567 (59.3)	60.0	11,291 (54.6)	48.8	–
Missing data	21 (0.0)	20 (0.0)	–	1 (0.0)	–	–
**Body mass index**						<0.001
<19	6,838 (2.8)	6,214 (2.8)	2.8	624 (3.0)	3.3	–
19 to 24	114,220 (46.8)	103,002 (46.1)	46.2	11,218 (54.3)	51.8	–
≥25	122,557 (50.2)	113,849 (51.0)	51.0	8,708 (42.1)	44.9	–
Missing data	432 (0.2)	320 (0.1)	–	112 (0.5)	–	–
**Trimester of LMC Registration**						<0.001
1	142,641 (58.5)	129,238 (57.9)	58.6	13,403 (64.9)	60.4	–
>1	99,302 (40.7)	92,050 (41.2)	41.4	7,252 (35.1)	39.6	–
Missing	2,104 (0.9)	2097 (0.9)	–	7 (0.0)	–	–
**Smoking Status**						<0.001
No	206,714 (84.7)	187,182 (83.8)	84.2	19,532 (94.5)	90.7	–
Yes	37,263 (15.3)	36,152 (16.2)	15.8	1,111 (5.4)	9.3	–
Missing data	70 (0.0)	51 (0.0)	–	19 (0.1)	–	–
**Pre-existing Diabetes and/or Hypertension**						0.465
No	242,327 (99.3)	221,848 (99.3)	99.3	20,479 (99.1)	99.3	–
Yes	1,720 (0.7)	1,537 (0.7)	0.7	183 (0.9)	0.7	–
**Gestational Age at Delivery (whole weeks)**						<0.001
37	13,486 (5.5)	12,113 (5.4)	5.4	1,373 (6.7)	6.3	–
38	37,299 (15.3)	32,941 (14.8)	14.8	4,358 (21.1)	19.3	–
39	65,707 (26.9)	59,524 (26.7)	26.7	6,183 (29.9)	28.5	–
40	78,583 (32.2)	72,744 (32.6)	32.5	5,839 (28.3)	30.1	–
41	41,882 (17.2)	39,222 (17.6)	17.5	2,660 (12.9)	14.3	–
42+	7,090 (2.9)	6,841 (3.1)	3.1	249 (1.2)	1.5	–

Table 2 presents the number and rates of adverse outcomes in total and by exposure group.

**Table 2:** Numbers and incidence rates of perinatal outcomes by 1,000 total and live births.

	Overall	Midwife-Led	Medical-Led
**Total Births**	244,047	223,385 (91.5%)	20,662 (8.5%)
**Live Births**	243,726	223,086	20,640
	***n* (rate)**	***n* (rate)**	***n* (rate)**
Perinatal related mortality*	437 (1.79)	410 (1.84)	27 (1.31)
Stillbirth*	321 (1.32)	299 (1.34)	22 (1.06)
Neonatal mortality**	116 (0.48)	111 (0.50)	5 (0.24)
Low Apgar**	2,283 (9.37)	2,183 (9.79)	100 (4.84)
Missing data (%)	20,356 (8.4%)	17,917 (8.3%)	2,439 (11.8%)
Intrauterine hypoxia/birth asphyxia/neonatal encephalopathy**	2,164 (8.88)	2,049 (9.18)	115 (5.57)
Birth-related asphyxia**	1,059 (4.35)	1,019 (4.57)	40 (1.94)
Intrauterine hypoxia**	1,003 (4.12)	935 (4.19)	68 (3.29)
Neonatal encephalopathy**	354 (1.45)	335 (1.50)	19 (0.92)
Small for gestational age**	22,984 (99.43)	21,107 (100.17)	1,877 (91.81)
Missing data (%)	12,579 (5.2%)	12,382 (5.6%)	197 (0.9%)

*Rate per 1,000 total births in specified group.

**Rate per 1,000 live births in specified group, excluding those missing data.

In the logistic regression model for perinatal mortality, after adjustment, the odds of death were higher for mothers who were smokers at registration, had a BMI ≥25, registered at a later trimester, or were nulliparous (Table 3). Adjusted odds of each of the morbidity-related outcomes were higher for mothers who were smokers at registration or were nulliparous, with smoking being particularly strongly related to SGA (OR 2.56; 95% CI: 2.46–2.66), and nulliparity with birth asphyxia/hypoxia/encephalopathy (OR 2.44; 2.22–2.67). Late trimester at registration was not independently associated with the two morbidity outcomes (included in these analysis) but was weakly associated with SGA (OR 1.11; 1.08–1.14). High BMI, by contrast, was associated with higher odds of morbidity outcomes (adjusted ORs were 1.27; 1.16–1.39 for birth asphyxia/hypoxia/encephalopathy and 1.29; 1.18–1.41 for low Apgar) but lower adjusted odds of SGA (OR 0.72; 0.70–0.74) (Tables 3 and 4).

**Table 3:** Adjusted ORs of PRM and birth asphyxia/hypoxia/neonatal encephalopathy in relation to maternal characteristics.

	ORs for Perinatal Outcomes (95% CI) with *p*-Value for Factor*					
	Perinatal Related Mortality			Birth asphyxia/hypoxia/neonatal encephalopathy		
	Crude	Adjusted**	*p*-Value (Adjusted)	Crude	Adjusted**	*p*-Value (Adjusted)
**Age**			0.785			0.026
<20	1.39 (1.00–1.94)	1.04 (0.73–1.49)		1.38 (1.19–1.60)	0.88 (0.75–1.04)	
20–35	1	1		1	1	
>35	0.97 (0.75–1.26)	1.10 (0.84–1.44)		0.91 (0.81–1.03)	1.15 (1.01–1.30)	
**Ethnicity**			0.399			<0.001
European	1	1		1	1	
Māori	1.27 (1.02–1.59)	0.90 (0.69–1.17)		1.17 (1.06–1.29)	1.02 (0.91–1.15)	
Pacific	1.53 (1.13–2.07)	1.13 (0.81–1.58)		0.94 (0.80–1.10)	0.77 (0.65–0.91)	
Asian	0.91 (0.61–1.35)	0.98 (0.65–1.48)		0.65 (0.53–0.79)	0.63 (0.51–0.77)	
Indian	1.58 (0.96–2.59)	1.56 (0.95–2.57)		1.16 (0.90–1.48)	1.06 (0.83–1.36)	
Other	0.90 (0.40–2.03)	0.90 (0.40–2.04)		0.99 (0.71–1.38)	0.90 (0.64–1.27)	
**NZ Deprivation Decile**			0.266			<0.001
1 to 2	1	1		1	1	
3 to 4	0.90 (0.62–1.31)	0.87 (0.60–1.27)		1.12 (0.94–1.33)	1.10 (0.92–1.31)	
5 to 6	1.20 (0.85–1.69)	1.12 (0.79–1.57)		1.54 (1.32–1.81)	1.51 (1.29–1.77)	
7 to 8	1.28 (0.92–1.78)	1.11 (0.80–1.56)		1.59 (1.37–1.86)	1.55 (1.32–1.81)	
9 to 10	1.59 (1.16–2.18)	1.26 (0.90–1.78)		1.66 (1.43–1.93)	1.60 (1.36–1.88)	
**Smoking**			<0.001			<0.001
Smoker	1.71 (1.37–2.14)	1.63 (1.27–2.09)		1.32 (1.19–1.47)	1.24 (1.10–1.40)	
Nonsmoker	1	1		1	1	
**BMI**			<0.001			<0.001
<19	0.84 (0.41–1.70)	0.80 (0.39–1.64)		0.78 (0.57–1.07)	0.81 (0.59–1.10)	
19–24	1	1		1	1	
25+	1.56 (1.28–1.90)	1.49 (1.21–1.83)		1.27 (1.17–1.39)	1.27 (1.16–1.39)	
**Pre-existing Diabetes and/or Hypertension**			0.446			0.258
Yes	1.97 (0.88–4.41)	1.41 (0.58–3.44)		1.52 (1.01–2.30)	1.27 (0.84–1.93)	
No	1	1		1	1	
**Trimester at Registration**			0.002			0.190
Trimester 1	1	1		1	1	
Trimester 2 or later	1.53 (1.27–1.85)	1.37 (1.13–1.67)		1.13 (1.04–1.23)	1.06 (0.97–1.16)	
**Parity**			<0.001			<0.001
Nulliparous	1.32 (1.09–1.59)	1.47 (1.20–1.81)		2.12 (1.95–2.31)	2.44 (2.22–2.67)	
Prima or multiparous	1	1		1	1	

*p-value from model Type III test for overall impact of factor (across all categories) on outcome.

**Adjusted for all other variables in the table.

**Table 4:** Adjusted ORs of low Apgar and small for gestational age in relation to maternal characteristics.

ORs for Perinatal Outcomes (95% CI) with *p*-Value for Factor*						
	Low Apgar			Small for Gestational Age (SGA)		
	Crude	Adjusted**	*p*-Value (Adjusted)	Crude	Adjusted**	*p*-Value (Adjusted)
**Age**			0.197			<0.001
<20 y	1.34 (1.16–1.55)	1.00 (0.85–1.17)		1.42 (1.35–1.49)	0.85 (0.81–0.90)	
20–35 y	1	1		1	1	
>35 y	0.95 (0.85–1.07)	1.12 (0.99–1.26)		0.84 (0.81–0.88)	1.12 (1.07–1.16)	
**Ethnicity**			0.374			<0.001
European	1	1		1	1	
Māori	1.17 (1.06–1.29)	1.02 (0.92–1.15)		1.62 (1.57–1.67)	1.32 (1.27–1.37)	
Pacific	1.10 (0.95–1.28)	0.93 (0.79–1.10)		1.00 (0.94–1.05)	1.13 (1.06–1.20)	
Asian	0.94 (0.80–1.11)	0.99 (0.84–1.17)		1.84 (1.76–1.93)	1.67 (1.59–1.75)	
Indian	1.15 (0.90–1.48)	1.13 (0.89–1.45)		3.62 (3.41–3.85)	3.58 (3.36–3.81)	
Other	1.29 (0.96–1.73)	1.30 (0.97–1.75)		1.45 (1.31–1.60)	1.51 (1.37–1.68)	
**NZ Deprivation Decile**			0.019			<0.001
1 to 2	1	1		1	1	
3 to 4	1.00 (0.85–1.17)	0.97 (0.83–1.14)		1.05 (1.00–1.11)	1.03 (0.98–1.09)	
5 to 6	1.21 (1.05–1.40)	1.15 (1.00–1.34)		1.16 (1.11–1.22)	1.09 (1.03–1.14)	
7 to 8	1.25 (1.08–1.43)	1.15 (1.00–1.33)		1.27 (1.21–1.33)	1.14 (1.08–1.20)	
9 to 10	1.33 (1.16–1.52)	1.19 (1.03–1.38)		1.51 (1.45–1.58)	1.30 (1.24–1.37)	
**Smoking**			<0.001			<0.001
Smoker	1.31 (1.18–1.45)	1.25 (1.11–1.41)		2.23 (2.16–2.30)	2.56 (2.46–2.66)	
Nonsmoker	1	1		1	1	
**BMI**			<0.001			<0.001
<19	1.06 (0.82–1.38)	1.03 (0.79–1.34)		1.81 (1.69–1.93)	1.58 (1.48–1.69)	
19–24	1	1		1	1	
25+	1.28 (1.18–1.40)	1.29 (1.18–1.41)		0.74 (0.72–0.76)	0.72 (0.70–0.74)	
**Pre-existing Diabetes and/or Hypertension**			0.940			<0.001
Yes	1.23 (0.76–1.98)	1.02 (0.63–1.65)		1.05 (0.90–1.23)	1.36 (1.16–1.60)	
No	1	1		1	1	
**Trimester at Registration**			0.792			<0.001
Trimester 1	1	1		1	1	
Trimester 2 or later	1.06 (0.97–1.15)	0.99 (0.91–1.08)		1.25 (1.22–1.29)	1.11 (1.08–1.14)	
**Parity**			<0.001			<0.001
Nulliparous	1.71 (1.58–1.86)	1.85 (1.70–2.03)		1.76 (1.71–1.80)	1.82 (1.76–1.87)	
Prima or multiparous	1	1		1	1	

*p-value from model Type III test for overall impact of factor (across all categories) on outcome.

**Adjusted for all other variables in the table.

Compared with mothers aged 20–35 y, age under 20 y was not associated with the morbidity-related outcomes but was associated with SGA. Compared with mothers aged 20–35 y, age greater than 35 y was associated with higher adjusted odds of the morbidity outcomes (ORs were 1.15; 1.01–1.30 for birth asphyxia/hypoxia/encephalopathy and 1.12; 0.99–1.26 for low Apgar) and SGA (OR 1.12; 1.07–1.16). Compared with other mothers, Māori and Pacific mothers were at increased crude odds of PRM; however, this association was not apparent after adjusting for the other covariates. Pacific and Asian mothers had lower adjusted odds of birth asphyxia/hypoxia/neonatal encephalopathy. Māori, Pacific, Asian, Indian, and Other mothers all had higher odds of SGA compared with other mothers. High deprivation (NZDep 9–10) was associated with higher adjusted odds of the morbidity-related outcomes and SGA, with ORs ranging from 1.19; (95% CI 1.03–1.38) to 1.60 (1.36–1.88). Mothers with pre-existing conditions were more likely to have SGA babies (OR 1.36; 1.16–1.60) (Tables 3 and 4).

Compared with midwife-led births, after adjusting for age, ethnicity, deprivation, BMI, smoking, parity, trimester of registration, and pre-existing conditions, there were nonstatistically significant lower odds of stillbirth (OR 0.86; 0.55–1.34), neonatal mortality (OR 0.62; 0.25–1.53), and PRM (OR 0.80; 0.54–1.19) among medical-led births—in all cases, confidence intervals were wide, and it was not possible to definitively say whether one model of care was associated with fewer deaths than the other. For medical-led births compared with midwife-led births, after adjusting for covariates, there were 48% lower odds of an Apgar score of less than seven at 5 min (OR 0.52; 0.43–0.64), 55% lower odds of birth-related asphyxia (OR 0.45; 0.32–0.62), 39% lower odds of neonatal encephalopathy (OR 0.61; 0.38–0.97), and 21% lower odds of intrauterine hypoxia (OR 0.79; 0.62–1.02) (Table 5). There was no difference in adjusted odds of being SGA between groups (OR 1.00; 0.95–1.05) (Table 5).

**Table 5:** Crude and adjusted ORs (with *p*-value) for all outcomes comparing medical-led to midwife-led care.

Outcome	OR (95% CI) and *p*-Value for Comparison		
	Crude (CI)	Adjusted (CI)***	*p*-Value (Adjusted)
**Perinatal Related Mortality** *	0.71 (0.48–1.05)	0.80 (0.54–1.19)	0.274
Stillbirth*	0.80 (0.52–1.23)	0.86 (0.55–1.34)	0.508
Neonatal mortality**	0.49 (0.20–1.19)	0.62 (0.25–1.53)	0.294
**Low Apgar (<7) at 5 min** **	0.51 (0.42–0.63)	0.52 (0.43–0.64)	<0.001
**Hypoxia/Asphyxia/Neonatal Encephalopathy** **	0.60 (0.50–0.73)	0.62 (0.51–0.75)	<0.001
Intrauterine hypoxia**	0.79 (0.61–1.01)	0.79 (0.62–1.02)	0.074
Birth-related asphyxia**	0.42 (0.31–0.58)	0.45 (0.32–0.62)	<0.001
Neonatal encephalopathy**	0.61 (0.39–0.97)	0.61 (0.38–0.97)	0.036
**Small for Gestational Age (<10th percentile for week)** **	0.91 (0.86–0.95)	1.00 (0.95–1.05)	0.931

*Denominator includes total births in the study population.

**Denominator includes total births in the study population minus those in which a stillbirth occurred.

***Adjusted for age, ethnicity, NZDep, BMI, smoking, parity, trimester of registration, and pre-existing hypertension and/or diabetes.

When we limited the analysis to low-risk mothers with a BMI of less than 25, nonsmokers at registration, and no pre-existing hypertension and/or diabetes, without exception the associations seen were slightly stronger than those for the whole cohort. For example, after adjusting for age, ethnicity, deprivation, parity, and trimester of registration, ORs for medical- compared with midwife-led births were as follows: for low Apgar score at 5 min, 0.48; 0.36–0.65, and for combined intrauterine hypoxia, birth-related asphyxia, and neonatal encephalopathy, 0.54; 0.40–0.73 (see S1 Table). Accounting for the potential impact of clustering of pregnancy outcomes by hospital (District Health Board [DHB]) resulted in failure to converge for mortality outcomes and results that were largely consistent with the main findings but with some movement towards the null for low Apgar (OR 0.60; 0.48–0.74) and combined intrauterine hypoxia, birth-related asphyxia, and neonatal encephalopathy (0.76; 0.62–0.94) (see S2 Table).

## Discussion

### Key Findings

Results from our study showed that after adjusting for likely confounding, medically led (i.e., having a medical LMC at first registration) births were associated with substantially lower odds of an Apgar score of less than seven at 5 min, birth-related asphyxia, and neonatal encephalopathy when compared with midwife-led births. Medical-led births were also associated with somewhat lower odds of stillbirth and neonatal mortality and intrauterine hypoxia; however, confidence intervals in these instances were wide and included the null. There was no association between model of care and being SGA after adjustments were made. The associations between other risk factors and the outcomes assessed in this study were all largely as expected [11,16–20].

### Strengths and Weaknesses

This is one of very few population-based studies based on individual data that have been able to compare rare adverse fetal and neonatal outcomes among births with autonomous midwife-led care compared with medical-led care. This study uses national level data and includes around 85% of all births occurring in New Zealand over the period of the study.

Given the different demographic and risk factor distribution between the study groups, the potential for confounding needs to be carefully considered. We are reassured that confounding was unlikely to account for the key findings for the following reasons. First, we used being SGA as a negative control to assess for residual and unmeasured confounding [13]. SGA was included as a negative control because it was likely to share a similar confounding structure as the other outcomes (see S1 Fig) but is unlikely to be influenced by model of care. Because of the independent and strong relationships between the potentially confounding covariates and this end point, we would expect to observe an association between model of care and SGA if that association was due solely to confounding by those covariates. In fact, once we adjusted for the covariates, there was no association between model of care and SGA (OR 1.00; 0.95–1.05), suggesting that either these covariates are not acting as confounders in an important way or that they have been adequately adjusted for.

Because BMI has a different relationship with SGA than the other adverse outcomes, we stratified the cohort by BMI (≥25 and <25) and found that the results in relation to model of care and adverse outcomes were similar for both BMI groups. One exception was PRM, in which ORs comparing medical-led to midwife-led births showed a greater protective effect for medical-led births in the normal BMI cohort; however, the estimates were imprecise on both counts, and the confidence intervals were substantially overlapping and included the null (S1 Table). We also reran the main analyses for lower-risk women (BMI less than 25, nonsmokers, and no pre-existing hypertension and/or diabetes) and found that in all cases the associations were stronger than for the full cohort (S1 Table). Finally, we estimated the magnitude of residual confounding that would be necessary to completely explain the associations demonstrated using a formula from Greenland [21]. This formula allows one to calculate the impact of confounding on an estimated association given the strength of association between the putative confounder and the dependent and independent variables. Assuming the prevalence of the unmeasured confounder(s) would be such to maximize the magnitude of confounding, to completely explain the findings relating to low Apgar score at 5 min or the combined intrauterine hypoxia/birth-related asphyxia/neonatal encephalopathy outcome, the ORs between the putative confounder(s) and the exposure or the outcome would have to be very strong (greater than five and greater than four, respectively). It is difficult to think of an unmeasured confounder or confounders that would meet these criteria.

The second potential limitation was the use of retrospective data that in some instances lacked detail and possibly accuracy and completeness. Our exposure groups were classified based on the registered LMC at first registration. We were unable to differentiate between midwives who provided all care themselves from those who had a level of medical input. Thus, our study assesses midwife-led care, not (necessarily) sole midwife care compared with medical-led care. There were a small number of women who had a different registered LMC at delivery than the one identified at registration, i.e., there were a small number of participants in the midwife-led group who were registered with a hospital or an obstetrician at time of delivery (*n* = 4,727, 1.9%). There was no means of ascertaining when the transfer of care occurred. To assess the impact of this, we removed all these participants and reran our logistic regression models; the results were unchanged.

It is possible that there was an under-reporting of some outcomes in the data. However, it is difficult to conceptualize any mechanism in which this would be differential in relation to the care received, so the effect of this would be to decrease study power rather than to introduce bias. The possible exception to this is Apgar score after 5 min, for which there were data missing in 8.3% of the midwife-led group and 11.8% of the medical-led group. If these missing data were more likely to be low Apgar scores, then the ORs would exaggerate the positive effect of medical-led births. However, it seems more likely (rather than less) that babies with low Apgar scores would have them recorded because of their clinical significance, so this seems an unlikely explanation for this specific finding.

While our ORs for mortality outcomes indicated a reduced risk for medical-led births, consistent with morbidity outcomes, confidence intervals were wide because of the rarity of this outcome. However, we used all available data, so these results still provide the best estimates given the data and sample size constraints that we had.

There have been very few individual studies published that compare midwife-led care with other models of maternity care that are sufficiently powered to look at major adverse events such as those included in this study. As mentioned earlier, a recent systematic review comparing midwife-led maternity care with other models of care concluded that women with midwife-led care were “less likely to experience intervention and more likely to be satisfied with their care with at least comparable adverse outcomes” [2]. However, for reasons outlined previously, the findings of this review cannot be generalized to a population context of completely autonomous midwives. Many of the analytical observational studies comparing midwife-led versus standard care models do not have sample sizes that are adequately powered for investigating rare, adverse outcomes [22–24]. One of the larger studies was performed by the Birthplace in England Collaborative Group, which compared outcomes of women with singleton, term pregnancies who had planned to receive care at home, in freestanding midwifery units, in alongside midwifery (joined to an obstetric unit) units, and in obstetric units [25]. They found that there was no difference in a composite end point of mortality and morbidity-related outcomes between groups, although there was an increase in risk of the composite mortality and morbidity outcome for women who planned a home birth compared with women planning on birthing in an obstetric unit (OR 1.59; 95% CI 1.01–2.52), which was higher still for low-risk nulliparous women (OR 2.80; 1.59–4.92). Similarly, a study from Stockholm that compared women birthing in a hospital birth center (with midwife-led care) with those birthing in hospitals under standard care found no overall difference in rates of perinatal mortality among those using the birthing center [26]. However, perinatal mortality rates among primiparous women were higher for those who delivered at a birth center compared with those who birthed in hospitals under standard care (OR 2.2; 1.3–3.9). A New Zealand-based analysis of outcomes of babies according to their planned place of birth showed that women who planned home births tended to be older, European, and multiparous and to have a BMI in a normal range; crude analysis showed that these women had lower rates of adverse outcomes compared with other women, but no adjustment for confounding characteristics was made [27].

Two studies have been conducted in the Netherlands that compare adverse perinatal outcomes between autonomous midwife- and medical-led care [28,29]. The first study concluded that babies of low-risk mothers under midwife-led care had increased risk of delivery-related death compared with babies of high-risk mothers under medical-led care (relative risk 2.33, 95% CI 1.12–4.83) [28]. The authors of this study were unable to adjust for known risk factors because of the use of aggregated data in which case information was collected without recording personal characteristics of the woman (to guarantee anonymity). A more recent study in the Netherlands (but one that used data from a similar period) found no difference in relative risk of intrapartum and neonatal mortality between births that started in midwife-led versus secondary obstetric-led care (relative risk 0.88, 95% CI 0.52–1.46) [29].

The results of the current study may raise questions about some aspects of the safety of the midwife-led model of care, at least in the New Zealand context. However, it is also very important that the findings are interpreted in context of research supporting the many positive aspects of midwife-led care, such as lower intervention rates and greater patient satisfaction [2]. It is also important to interpret the findings in the context of New Zealand’s overall birth outcomes in comparison to other countries. The 2011 perinatal mortality rate (occurring at >24 wk gestation) in New Zealand (6.7) was similar to that of the United Kingdom, lying between the rates in Ireland (6.1) and England and Wales (7.5). Using a slightly different definition (deaths occurring at >20 wk gestation), New Zealand had a largely similar perinatal mortality rate at 10.6 deaths per 1,000 births compared to Australia’s at 9.8 deaths per 1,000 births [17]. This is reassuring in that it suggests that in absolute terms New Zealand’s maternity system is still internationally comparable in terms of adverse outcomes; however, it does not preclude the possibility that avoidable adverse outcomes are occurring, potentially both in New Zealand and in other countries.

### Recommendations and Further Research

There is a need to understand the reasons for the apparent excess of adverse outcomes in midwife-led deliveries in New Zealand. Despite a radical change in the way maternity care was delivered, there has never been a full and proper evaluation to ensure the maternity system in New Zealand is safe. This should be a major priority both for New Zealand and other countries with current or prospective midwife-led systems. Given the positive findings of the Cochrane review relating to midwife-led care, it may well be that midwife-led care is optimal within the context of well-organized systems [2]. However, there is an urgent need to establish which aspects of those systems potentially make that care more, or less, safe. This might include an evaluation of different approaches to training maternity care professionals, the impact of the level of collaboration between midwives and doctors, the triaging process in allocating midwife-led versus medical-led care, if there are any differences in risk of adverse outcomes relating to rurality or access to secondary services in midwife-led births, and the level of organization around antenatal care.

## Supporting Information

S1 Analysis Plan Analysis plan. (DOCX)

S1 Checklist STROBE statement. (DOC)

S1 Fig Directed acyclic graph. (TIF)

S1 Table Adjusted ORs for primary outcomes comparing medical-led and midwife-led care for stratified cohorts. (DOCX)

S2 Table Fully adjusted ORs for main outcomes comparing medical-led with midwife led care, clustered by DHB. (DOCX)

S3 Table Distribution of birthweight tenth percentile by gestational age (from all live births in current data with birthweight data available), as used for calculation of SGA. (DOCX)

## Abbreviations

BMI: body mass index
DHB: District Health Board
GP: general practitioner
LMC: lead maternity carer
NMDS: National Minimum Dataset
NZDep: New Zealand Deprivation Index
OR: odds ratio
PRM: perinatal related mortality
SGA: small for gestational age
STROBE: Strengthening the Reporting of Observational Studies in Epidemiology
