# Supplementary material for: A Comparison of Midwife-Led and Medical-Led Models of Care and Their Relationship to Adverse Fetal and Neonatal Outcomes: A Retrospective Cohort Study in New Zealand
Source: PLoS Med. 2016 Sep 27;13(9):e1002134. doi: 10.1371/journal.pmed.1002134 (PMC5038958; doi:10.1371/journal.pmed.1002134)
Supplement: S1 Analysis Plan — (DOCX) [file pmed.1002134.s001.docx]

**What impact does the New Zealand maternity care model have on fetal and neonatal outcomes? A retrospective cohort study comparing exclusive midwife led care with a shared care model.**

Background

New Zealand has a maternity system that has undergone a number of changes in the past twenty years that have moved it away from a medical model of care and towards a midwifery model [18]. The changes have been the subject of scrutiny and controversy focusing on questions related to the safety of the system [3, 5, 6, 7, 15, 16, 17, 20, 22]. Despite this, there has been little in the way of systematic evaluation that specifically relates to the safety of the maternity system [8, 10-14, 19].

New Zealand’s maternity system is based on a midwife-led care model, whereby midwives practice with a level of autonomy higher than most of their overseas counterparts [9, 21, 23]. International literature has generally supported the safety of the midwife-led care model [4]. However this does not provide sufficient evidence to support the safety of the New Zealand system, as in many countries where the international literature was based midwives have less autonomy than they do in New Zealand; so even in midwife-led births there is some medical input. The uniqueness of our system means that evaluation of the safety of New Zealand based midwife-led care needs to be addressed within New Zealand.

Along with the lack of New Zealand based research, impetus for research in this area has been provided by a recently published Dutch study [1]. Holland has a maternity system that is similar to ours in that midwives practice with a high level of autonomy; therefore evaluation in a Dutch context is relevant to us. The Dutch study found, most significantly, that “*infants of pregnant women at low risk whose labour started in primary care under the supervision of a midwife had a significant higher risk of delivery related perinatal death than infants of pregnant women at high risk whose labour started in secondary care under the supervision of an obstetrician… relative risk 2.33*” [1, p. 1]. Given the similarities between the Dutch maternity system and ours, this is a concerning finding in the New Zealand as well as Dutch context.

In contrast, further incentive for addressing the lack of New Zealand based research is provided with the demonstrated value of midwifery led care. Consumer satisfaction surveys have reported high levels of support for our current model [6]. The most recent (2011) survey reported on 3225 women; 89% of these women reported feeling satisfied or very satisfied with the care they received from their LMC. This is encouraging in terms of midwife-led care as three quarters of respondents were registered with a self-employed midwife as their LMC [6].

However, the media, consumer and professional opposition may have led to a decrease in women’s confidence of the system and has been reported as being discouraging to midwives [2]. Midwives have not had an evidence base to defend the current model, which has exacerbated the critique. If evidence can be produced that provides support for the safety of the current maternity system, then this can be used by midwives to defend their profession, and to generate support, and increase consumer confidence.

If however the study finds similar results to the Dutch study, in that midwife-led care leads to poorer outcomes for babies compared with medical care, this evidence can be used as incentive to generate further research that focuses on the reasons for this, with the aim to alleviate the causes and improve outcomes for babies.

Ultimately a safer maternity system that supports the best possible outcomes for babies is an important goal. Midwifery care is an essential part of our maternity system and it is essential for its continuation that it be based on evidence with its safety ensured.

Aim

This study aims to evaluate key aspects of the safety of the New Zealand maternity system. Specifically we will investigate serious fetal and neonatal outcomes that are potentially related to care provider.

Objectives

i) To review the literature regarding the safety of midwife-led care models within New Zealand and internationally.

ii) To demonstrate what data is available for the purpose of reviewing safety related fetal and neonatal outcomes in relation to maternity care provider, within New Zealand.

iii) To calculate and compare (where relevant) the incidence of potentially avoidable serious early neonatal morbidity and perinatal mortality, overall, and 1) in women whose LMC is reported as being a midwife where there is no evidence of medical input, 2) in women whose LMC is reported as being a midwife where there is evidence of medical input and 3) in women whose LMC is a general practitioner or obstetrician.

Inclusion criteria

- Singleton pregnancy
- Delivered during a specified time period (likely to be 1^st^ January 2006 and 1^st^ January 2010, The final inclusion period will be based on DHB birthing numbers and required sample size)
- Birthed at one of six specified District Health Boards
- Equal to or greater than 37 weeks gestation
- No identified major fetal or neonatal congenital, chromosomal, or metabolic abnormalities.

The sample size has been determined based on power calculations using the rarest outcome in the study, intrapartum fetal death. Sample size has been calculated using the Fleiss method with continuity correction in OpenEpi. Outcome rates have been estimated based on the Dutch Perinatal Mortality Study. An adequately powered simple study requires a sample size of 48 878. However, we need to increase the sample size because we wish to adjust the results for several major confounders particularly maternal age, antenatal risk, ethnicity, deprivation and parity of the mother. Furthermore, we wish to perform some subgroup analysis. For these reasons, we plan to double the sample size.

Exposure

- Reference group

Women who are reported to have a midwife as Lead Maternity Carer at booking and at admission in labour and where there is no evidence of medical input.

- Comparison groups

Women who are reported to have a midwife as LMC with evidence of medical input. Women will be included in this group if they have had one or more secondary care consultations or if they have had any antenatal admissions to hospital.

- Women who have had a General Practitioner, private obstetrician, or secondary care team as their LMC.

Outcomes

- Antepartum stillbirth: Antepartum stillbirth rates will be calculated as the number of fetal deaths that occur before the onset of labour divided by the total number of births in our cohort.
- Intrapartum mortality: Intrapartum mortality rates will be calculated as the number of fetal deaths that occur after the onset of labour but before delivery divided by the total number of births in our cohort, not including antenatal still births.
- Early neonatal mortality: Early neonatal mortality rates will be calculated as the number of early neonatal deaths, that is deaths that occur post-delivery and within seven days of life, divided by total number of births in our cohort, not including antenatal still births or babies who have died in the intrapartum period.
- Neonatal admission to a specialist neonatal unit: Neonatal admission rates will be calculated as the number of babies admitted to a specialist neonatal unit within the first seven days of life divided by the total number of births in our cohort but not including antenatal stillbirths, or babies dying intrapartum, or in the early neonatal period.
- Low Apgar score: Rates of low Apgar score will be calculated as the number of babies born with an Apgar score at five minutes post-delivery of below seven, divided by total number of births in our cohort, not including antenatal still births or babies who have died in the intrapartum period.
- Birth related asphyxia: Rates of birth related asphyxia will be calculated based on the number of babies identified with the condition by ICD-10-AM codes P210, P211, P219, divided by total number of births in our cohort, not including antenatal still births or babies who have died in the intrapartum period.
- Intrauterine hypoxia: Rates of intrauterine hypoxia will be calculated based on the number of babies identified with the condition by ICD-10-AM codes P200, P201, P209 divided by total number of births in our cohort, not including antenatal still births or babies who have died in the intrapartum period.
- Hypoxic ischaemic encephalopathy: Rates of hypoxic ischaemic encephalopathy will be calculated based on the number of babies identified with the condition by ICD-10-AM code P916, divided by total number of births in our cohort, not including antenatal still births or babies who have died in the intrapartum period.
- Low birth weight: Where the birth weight was recorded as less than 2500 grams. This variable was included as a negative control outcome in order to determine whether adjustment for confounding for the main outcomes was likely to have been sufficient. This is on the assumption that model of care is unlikely to substantially affect birth weight directly, but similar confounders may affect the relation between low birth weight, model of care and the outcomes

Covariates

The study will collect a number of maternal variables that may be potential confounders or mediators. These include:

- Maternal age: 12-18 yrs; 19-25 yrs; 26-35 yrs; 36-40 yrs and 40+.
- Ethnicity: NZ European, Māori, Pacific Island, Asian, Other.
- Deprivation: NZ Dep quartiles.
- Parity before delivery: 0, 1, 2, 3, 4+.
- Gestation at delivery: 37/40-38/40; 39/40-40/40; 41/40; 42+ weeks.
- Location of delivery: Primary birthing unit, secondary or tertiary hospital.
- Mode of delivery: Normal vaginal delivery, instrumental delivery, caesarean section.
- Pre-existing diabetes and/or hypertension: yes, no.

Analysis

- We will first calculate incidence rates (and 95% confidence intervals) of all outcomes for all groups combined and then for each group separately.
- We will then develop logistic regression models to calculate odds ratios (and 95% confidence intervals) for each major outcome comparing women receiving shared compared with midwife only care. The models will include potentially confounding patient characteristics such as age, parity, ethnicity and presence of pre-existing conditions.

Available data

1. EXPOSURE

For classifying the exposure status LMC data will be collected from individual DHBs. This category is the only one not available from the MOH who only hold LMC data for around 70% of women.

It is difficult to know how robust/complete the DHB data is before accessing it. Generally it is entered by clinicians. The available categories are as follows:

- LMC data:

LMC at booking

LMC at admission in labour

Labour care provided by*

Delivered by

Labour and delivery assisted by

*The last three are not collected by all the DHBs.

- Data to classify ‘medical input’:

A woman will be classified as having medical input if she has had antenatal secondary care consultations. This data is held by the MOH and comes from the National Non- Admitted Patient Collection. We will analyse the number of secondary care consultations and the type of consultation. The following codes are relevant:

| W03002 | First obstetric consults |
| --- | --- |
| W03003 | Subsequent obstetric consults |
| W03005 | Amniocentesis |
| W03006 | Chorion villis sampling |
| W03007 | Rhesus Clinics - multidisciplinary clinics |
| W03008 | Maternity foetal medicine clinics - multidisciplinary clinics |
| W03009 | Foetal medicine / anomalies clinics - multidisciplinary clinics |

Some of these categories are more likely to be associated with greater obstetric input; number of consults is also relevant. For example a woman who has one consult recorded under amniocentesis and no further consults likely had a negative test and limited obstetric input in her care. Sub-category analysis will therefore be necessary.

The number of obstetric related hospital admissions within the pregnancy is also available from the NMDs. These data are not detailed and we will be making the assumption that if a patient has been admitted to hospital she will have had some level of secondary care input. Again number of admissions is relevant and sub-category analysis necessary.

1. COVARIATES

All covariate data will be collected from the MOH. The covariates listed above are routinely collected, after hospital discharge, as part of the National Minimum Dataset (NMDS).

1. OUTCOMES

Outcomes are also collected from the MOH’s NMDS. We may need to use the Perinatal Maternal Mortality Review Committee Data to ascertain antepartum vs. intrapartum stillbirth as MOH data may not be sufficiently detailed.

**Reference**

[1] Evers A, Brouwers H, Hukkelhoven C, Nikkels P, Boon J, Egmond-Linden A, Hillegersberg J, Snuif Y, Sterken-Hooisma S, Bruinse H, & Kwee A (2010) ‘Perinatal mortality and severe morbidity in low and high risk term pregnancies in the Netherlands: Prospective cohort study’ *British Medical Journal, 341; c*5639.

[2] Guilliland K (2008) ‘Response to Media’ Retrieved 20^th^ May 2011 from <http://www.midwife.org.nz/index.cfm/3,223,485/dominion-post-opinion-piece-08.pdf>

[3] Haines L (2009) ‘Another unfortunate experiment?’ *The* *New Zealand Listener, 3586.*

[4] Hatem M, Sandall J, Devane D, Soltani H, Gates S (2009) ‘Midwife-led versus other models of care for childbearing women (Review)’ The Cochrane Collection: Wiley.

[5] Health Select Committee (2010) ‘Petition 2008/23 of Jennifer Maree Hooper; Report of the Health Committee’, Wellington: House of Representatives, available: [http://www.parliament.nz/NR/rdonlyres/550C0E33-82EB-4186-911E7DCE5F684689/163094/DBSCH_SCR_4887_Petition200823ofJenniferMareeHooper.pdf](http://www.parliament.nz/NR/rdonlyres/550C0E33-82EB-4186-911E-7DCE5F684689/163094/DBSCH_SCR_4887_Petition200823ofJenniferMareeHooper.pdf) [accessed March 24^th^ 2011]

[6] Ministry of Health (2012) ‘Maternity consumer surveys 2011’ Ministry of Health: Wellington.

[7] Ihaka J (2011) ‘Grieving parents push for midwifery changes’ New Zealand Herald.

[8] Kutinova A (2008) ‘Midwifery in New Zealand: Government policies, provider choice, and health outcomes. Department of Economics: Canterbury University.

[9] NHS (2009) ‘Maternity Statistics 2009’ retrieved 20^th^ May 2011 from: <http://www.ic.nhs.uk/statistics-and-data-collections/hospital-care/maternity/nhs-maternity-statistics-england--2009-10>

[10] Ministry of Health (1999) ‘Report on Maternity’ Ministry of Health: Wellington

[11] Ministry of Health (2000-2001) ‘Report on Maternity’ Ministry of Health: Wellington

[12] Ministry of Health (2002) ‘Report on Maternity: Newborn and Women’s Health’ Ministry of Health: Wellington.

[13] Ministry of Health (2003) ‘Report on Maternity: Newborn and Women’s Health’ Ministry of Health: Wellington.

[14] Ministry of Health (2004) ‘Report on Maternity: Newborn and Women’s Health’ Ministry of Health: Wellington.

[15] Newton K (2010) ‘Call for review of ‘near-miss’ births’ The Southland Times.

[16] New Zealand Herald (2006) ‘Midwife faces charge of manslaughter’

[17] New Zealand Herald (2011) ‘Midwife blamed for baby’s death’

[18] Pairman S, Pincombe J, Thorogood C, & Tracy S (2006) ‘Midwifery: Preparation for Practice’. Elsevier: Australia.

[19] Perinatal and Maternal Mortality Review Committee (PMMRC) (2010) ‘Perinatal and maternal mortality in New Zealand 2008’ PMMRC: Wellington.

[20] Royal College of Obstetricians and Gynaecologists (2008) ‘Maternity Services Review’ RANZCOG

[21] The Australian Health Workforce Advisory Committee (2002) ‘The Midwifery Workforce in Australia’ available: <http://www.ahwo.gov.au/documents/Publications/2002/The%20midwifery%20workforce%20in%20Australia.pdf>

[accessed June 24^th^ 2011]

[22] The Good Fight (2009) ‘Submission to the Health Select Committee on a Maternity System Review/ Inquiry in Response to the Petition from Jenn Hooper’, available: <http://www.thegoodfight.co.nz/Health_Select_Committee_Submission.html> [accessed March 24^th^ 2011]

[23] Wagner S (2006) ‘Childbirth in the USA: How a broken maternity system must be fixed to put women and children first’. University of California Press: California.

**APPENDIX 1: DIRECTED ACYCLIC GRAPH DEMONSTRATING RELATIONSHIPS BETWEEN EXPOSURES, COVARIATES, AND OUTCOMES.**

BIRTH WEIGHT

AGE, ETHNICITY, SEP, SMOKING, BMI, PARITY, TRIMESTER OF REGISTRATION, PRE-EXISTING CONDITIONS

MODEL OF MATERNITY CARE

PERINATAL MORTALITY AND MORBIDITY
