# Supplementary figures and images for: A Comparison of Midwife-Led and Medical-Led Models of Care and Their Relationship to Adverse Fetal and Neonatal Outcomes: A Retrospective Cohort Study in New Zealand
Source: PLoS Med. 2016 Sep 27;13(9):e1002134. doi: 10.1371/journal.pmed.1002134 (PMC5038958; doi:10.1371/journal.pmed.1002134)

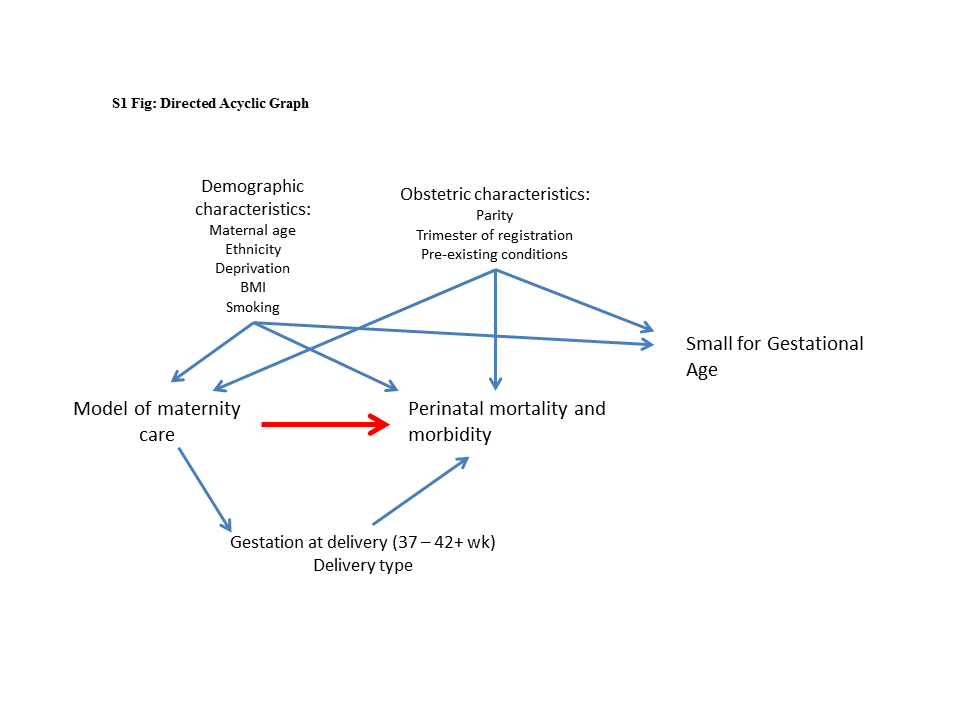

Supplement: S1 Fig — (TIF) [file pmed.1002134.s003.tif]
