# Supplementary material for: A Comparison of Midwife-Led and Medical-Led Models of Care and Their Relationship to Adverse Fetal and Neonatal Outcomes: A Retrospective Cohort Study in New Zealand
Source: PLoS Med. 2016 Sep 27;13(9):e1002134. doi: 10.1371/journal.pmed.1002134 (PMC5038958; doi:10.1371/journal.pmed.1002134)
Supplement: S1 Table — (DOCX) [file pmed.1002134.s004.docx]

**S1 Table**

| **Table S1** Adjusted odds ratios for primary outcomes comparing medical-led and midwife-led care for stratified cohorts | | | | | | |
| --- | --- | --- | --- | --- | --- | --- |
| **Outcome** | **Odds Ratio** | | | | | |
|  | **BMI <25 Cohort** | **p-value (Adjusted)** | **BMI >= 25 cohort** | **p-value (Adjusted)** | **Low Risk^1^ cohort** | **p-value (Adjusted)** |
| Perinatal related mortality* | 0.65 (0.34-1.25) | 0.193 | 0.93 (0.57-1.54) | 0.790 | 0.71 (0.37-1.36) | 0.288 |
| Low Apgar (<7) at five minutes** | 0.50 (0.38-0.67) | <0.001 | 0.54 (0.40-0.73) | <0.001 | 0.48 (0.36-0.65) | <0.001 |
| Hypoxia/ asphyxia/ NEC** | 0.56 (0.42-0.74) | <0.001 | 0.67 (0.51-0.87) | 0.003 | 0.54 (0.40-0.73) | <0.001 |
| ^1^Cohort includes all study participants with a BMI <25, non-smokers at registration and no identification of pre-existing hypertension and/or diabetes. | | | | | | |
| *Adjusted for age, ethnicity, NZ Dep, smoking, parity, trimester of registration, and pre-existing hypertension and/or diabetes. Denominator includes all births. | | | | | | |
| **Adjusted for age, ethnicity, NZ Dep, parity, and trimester of registration. Denominator includes all live births. | | | | | | |
