# Supplementary material for: A Comparison of Midwife-Led and Medical-Led Models of Care and Their Relationship to Adverse Fetal and Neonatal Outcomes: A Retrospective Cohort Study in New Zealand
Source: PLoS Med. 2016 Sep 27;13(9):e1002134. doi: 10.1371/journal.pmed.1002134 (PMC5038958; doi:10.1371/journal.pmed.1002134)
Supplement: S2 Table — (DOCX) [file pmed.1002134.s005.docx]

**S2 Table**

| **Table S2** Fully adjusted odds ratios for main outcomes comparing medical-led with midwife led care, clustered by DHB | | | | |
| --- | --- | --- | --- | --- |
| **Outcome** | **Adjusted (CI)*** | **p-value (original model)** | **+Cluster DHB**** | **p-value (cluster DHB model)** |
| **Perinatal related mortality***** | 0.80 (0.54-1.19) | 0.274 | n/a | n/a |
| **Low Apgar (<7) at five minutes****** | 0.52 (0.43-0.64) | <0.001 | 0.60 (0.48-0.74) | <0.001 |
| **Hypoxia/ asphyxia/ neonatal encephalopathy****** | 0.62 (0.51-0.75) | <0.001 | 0.76 (0.62-0.94) | 0.010 |
| **Small for Gestational Age****** | 1.00 (0.95-1.05) | 0.931 | 1.03 (0.97-1.09) | 0.287 |
| *Fully adjusted model from Table 5 in paper | | | | |
| ** Including DHB as a random effect (random intercept model by DHB) – n/a indicates that model failed to converge for mortality. | | | | |
| *** Adjusted for age, ethnicity, NZ Dep, smoking, parity, trimester of registration, and pre-existing hypertension and/or diabetes. Denominator includes all births. | | | | |
| ****Adjusted for age, ethnicity, NZ Dep, parity, and trimester of registration. Denominator includes all live births. | | | | |
