# Supplementary material for: A Comparison of Midwife-Led and Medical-Led Models of Care and Their Relationship to Adverse Fetal and Neonatal Outcomes: A Retrospective Cohort Study in New Zealand
Source: PLoS Med. 2016 Sep 27;13(9):e1002134. doi: 10.1371/journal.pmed.1002134 (PMC5038958; doi:10.1371/journal.pmed.1002134)
Supplement: S3 Table — (DOCX) [file pmed.1002134.s006.docx]

**S3 Table**

**Table S3-1. Distribution of birthweight tenth-percentile by gestational age (from all live births in current data with birthweight data available), as used for calculation of SGA.**

| Gestational age (weeks) | 10th percentile (grams) |
| --- | --- |
| 37 | 2490 |
| 38 | 2740 |
| 39 | 2935 |
| 40 | 3065 |
| 41 | 3195 |
| 42+ | 3230 |

**Table S3-2. Distribution of birthweight tenth-percentile by gestational age (from all live births in current data with birthweight data available), as used for calculation of SGA, plus ethnic specific 10th percentiles (calculated from McCowan & Stewart, 2004: data only available from 38-41 weeks.)**

| Gestational age (weeks) |  | 10th percentile (grams) | | | | | | | | |
| --- | --- | --- | --- | --- | --- | --- | --- | --- | --- | --- |
|  |  | Current Study* |  | McCowan & Stewart 2004**  (average of Male and Female infant 10th percentiles) | | | | | | |
|  |  | All infants |  | European | Māori | Samoan | Tongan | Chinese | Indian | Other |
|  |  |  |  |  |  |  |  |  |  |  |
| 37 |  | 2490 |  | - | - | - | - | - | - | - |
| 38 |  | 2740 |  | 2735 | 2665 | 2860 | 2910 | 2720 | 2455 | 3710 |
| 39 |  | 2935 |  | 2895 | 2825 | 3020 | 3065 | 2880 | 2615 | 2870 |
| 40 |  | 3065 |  | 3040 | 2970 | 3165 | 3215 | 3025 | 2765 | 3020 |
| 41 |  | 3195 |  | 3180 | 3110 | 3305 | 3355 | 3165 | 2900 | 3160 |
| 42 |  | 3230 |  | - | - | - | - | - | - | - |
|  |  |  |  |  |  |  |  |  |  |  |

******* Tenth percentile birthweight for current dataset (restricted to live births)

**** Average of Male and Female 10th percentiles from:*** McCowan , L, & Stewart, AW. (2004) Term birthweight centiles for babies from New Zealand’s main ethnic groups ***Australian and New Zealand Journal of Obstetrics and Gynaecology; 44: 432–435***
